# Cellular senescence is associated with osteonecrosis of the femoral head while mesenchymal stem cell conditioned medium inhibits bone collapse

**DOI:** 10.1038/s41598-024-53400-w

**Published:** 2024-02-09

**Authors:** Masanori Okamoto, Hiroaki Nakashima, Kiyoshi Sakai, Yasuhiko Takegami, Yusuke Osawa, Junna Watanabe, Sadayuki Ito, Hideharu Hibi, Shiro Imagama

**Affiliations:** 1 Department of Orthopaedic Surgery, Nagoya University Graduate School of Medicine, 65 Tsurumai, Shouwa-ku, Nagoya, Aichi 466-8560 Japan; 2 Department of Oral and Maxillofacial Surgery, Nagoya University Graduate School of Medicine, 65 Tsurumai, Shouwa-ku, Nagoya, Aichi 466-8560 Japan

**Keywords:** Mesenchymal stem cells, Cell death, Senescence, Osteoarthritis

## Abstract

Osteonecrosis of the femoral head (ONFH) is a type of ischemic osteonecrosis that causes pain, loss of function, and femoral head collapse. Here, we analyzed samples of femoral heads excised from patients with ONFH to clarify the relationship between ischemic osteonecrosis and cellular senescence. X-gal staining was strong and p16INK4a-positive cells were abundant in the transitional region of ONFH. The β-galactosidase-positive cells in the transitional region were also positive for nestin, periostin, or DMP-1. In contrast, no β-galactosidase-positive cells were detected in the healthy region. The senescence-associated p16INK4a, p21, and p53 were upregulated in ONFH tissue. We also examined and analyzed a mouse ischemic femoral osteonecrosis model in vivo to verify the association between ONFH and cellular senescence. Human mesenchymal stem cell-conditioned medium (MSC-CM) was administered to determine its therapeutic efficacy against cellular senescence and bone collapse. MSC-CM reduced the number of senescent cells and downregulated the aforementioned senescence-related genes. It also decreased the number of empty lacunae 4 weeks after ischemia induction and promoted bone formation. At 6 weeks post-surgery, MSC-CM increased the trabecular bone volume, thereby suppressing bone collapse. We conclude that cellular senescence is associated with ONFH and that MSC-CM suppresses bone collapse in this disorder.

## Introduction

Osteonecrosis of the femoral head (ONFH) is bone death associated with circulatory disruption. ONFH develops when the bone head collapses. This disorder eventually progresses to secondary osteoarthritis^1^. Epidemiological studies conducted in Asia have indicated an ONFH prevalence was between 28.91 and 725 per 100,000 population^2,3^, and in the United States, nearly 20,000 new cases are diagnosed annually^4^. ONFH occurs mostly in younger patients^4,5^. Risk factors for the development of ONFH include long-term alcohol and corticosteroid use^6,7^. Its pathogenesis is associated with dyslipidemia, thrombosis, cell death, oxidative stress, and damage-associated molecular patterns (DAMPs) but has not yet been fully elucidated^8–14^.

Non-surgical ONFH treatment significantly reduces patient quality of life (QoL) as it causes pain, impaired mobility, and progressive joint deformity^1,4^. Hip‐preserving surgical treatments are invasive, technically difficult, and often result in unsatisfactory outcomes such as failure to ensure long-term protection of the femoral head^15–17^. The only currently available treatment is total hip arthroplasty (THA) or replacement of the osteonecrotic area with an artificial hip joint. Nevertheless, this procedure is also invasive. Moreover, multiple surgeries may be required for younger patients as the prostheses wear out with use. Hence, a therapeutic agent that efficaciously treats ONFH with minimal invasion is required^18,19^.

Healthy cells may become senescent in response to irreparable DNA damage caused by various factors^20^. Senescent cells acquire the senescence-associated secretory phenotype (SASP) wherein they release proinflammatory cytokines, chemokines, and extracellular matrix-degrading enzymes^21,22^. Cellular senescence and SASP are associated with arteriosclerosis, type 2 diabetes mellitus (T2DM), renal failure, osteoarthritis, osteoporosis, and bisphosphonate-related osteonecrosis of the jaw (BRONJ)^23–30^. However, the involvement of cellular senescence in ONFH or ischemic osteonecrosis has not been investigated. The association between osteonecrosis and cellular senescence was reported for a BRONJ model^29^. A rat drug-induced BRONJ model was developed by administering zoledronic acid to the animals and extracting their teeth. β-galactosidase-positive cells were detected in the tooth sockets and the bone was exposed^29^. For this reason, it was postulated that ischemic osteonecrosis or ONFH may also be associated with cellular senescence. In a preliminary study, we stained samples of femoral heads from patients with ONFH and detected numerous senescent cells. Thus, we began to investigate the association between ONFH and cellular senescence.

“Senotherapy” is a therapeutic approach that involves targeting senescent cells to delay senescence and limit dysfunction. One procedure is in this regard is the pharmacological elimination of senescent cells, referred to as senolytics. An alternative to senolytics, is the senomorphic approach, which involves the alleviation of cellular senescence-related phenotypes without eliminating senescent cells^26,31^. In elderly mice, MSC-CM has been shown to reduce the percentage of senescence-associated (SA)-β-galactosidase-positive bone marrow-derived MSCs and to downregulate *p16INK4a*^32^, and has also been found to suppress chronic hyperglycemia-induced cellular senescence in fibroblasts^33^. Thus, MSC-CM also functions as a senomorphic medium, in that it suppresses senescent phenotypes^32,33^. In addition, in a BRONJ model, the administration of extracellular vesicles purified from MSC-CM eliminated β-galactosidase-positive cells and prevented the senescence of cells involved in wound healing and bone formation, as well as BRONJ^29^. On the basis of the foregoing findings, we speculated that MSC-CM would also have therapeutic efficacy against ONFH.

In the present study, we showed that senescent cells accumulated in the transitional regions of human ONFH and a mouse ischemic osteonecrosis model. In the latter, MSC-CM administration reduced cellular senescence and prevented bone collapse by promoting bone formation. To the best of our knowledge, this work is the first translational study to (1) confirm the presence of senescent cells in both human ONFH samples and a mouse ischemic osteonecrosis model, and (2) show that MSC-CM administration can reduce post-ONFH bone collapse associated with cellular senescence.

## Results

### Senescent cells accumulate in the transitional region of human osteonecrosis of the femoral head (ONFH)

**Figure 1:**
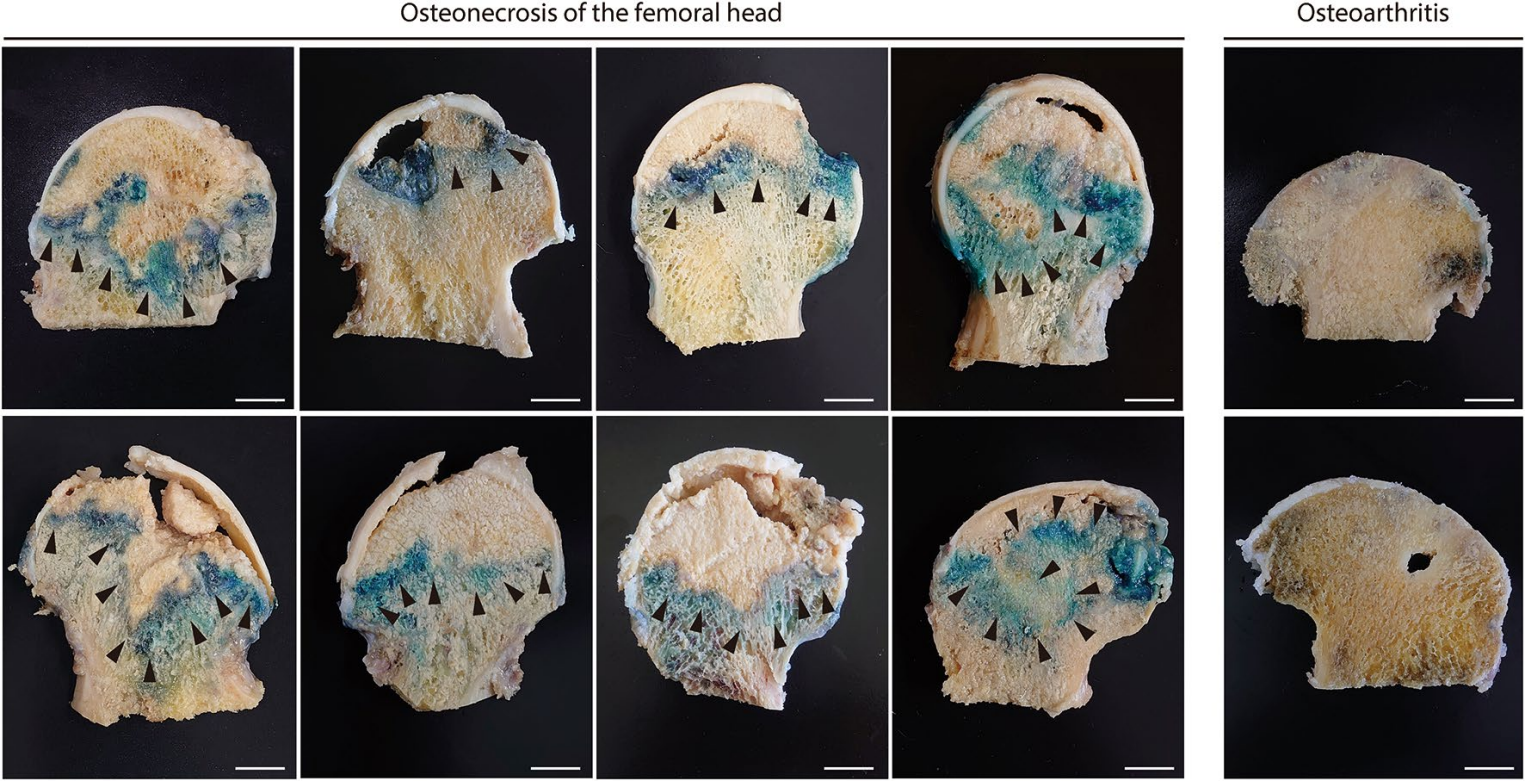
Senescent cells zonally accumulated in the femoral heads of patient with ONFH. Photograph of the coronal section of a human femoral head stained with X-gal. Strong X-gal staining (blue) was observed in the transitional region, which appeared as a serpentine band indicated by black arrows, but was seldom seen in the necrotic or healthy regions of the femoral heads in patients with osteonecrosis of the femoral head (ONFH). X-gal staining was seldom detected in osteoarthritis (OA). The femoral heads of eight patients with ONFH and two patients with OA were stained. Scale bar = 10 mm.

**Figure 2:**
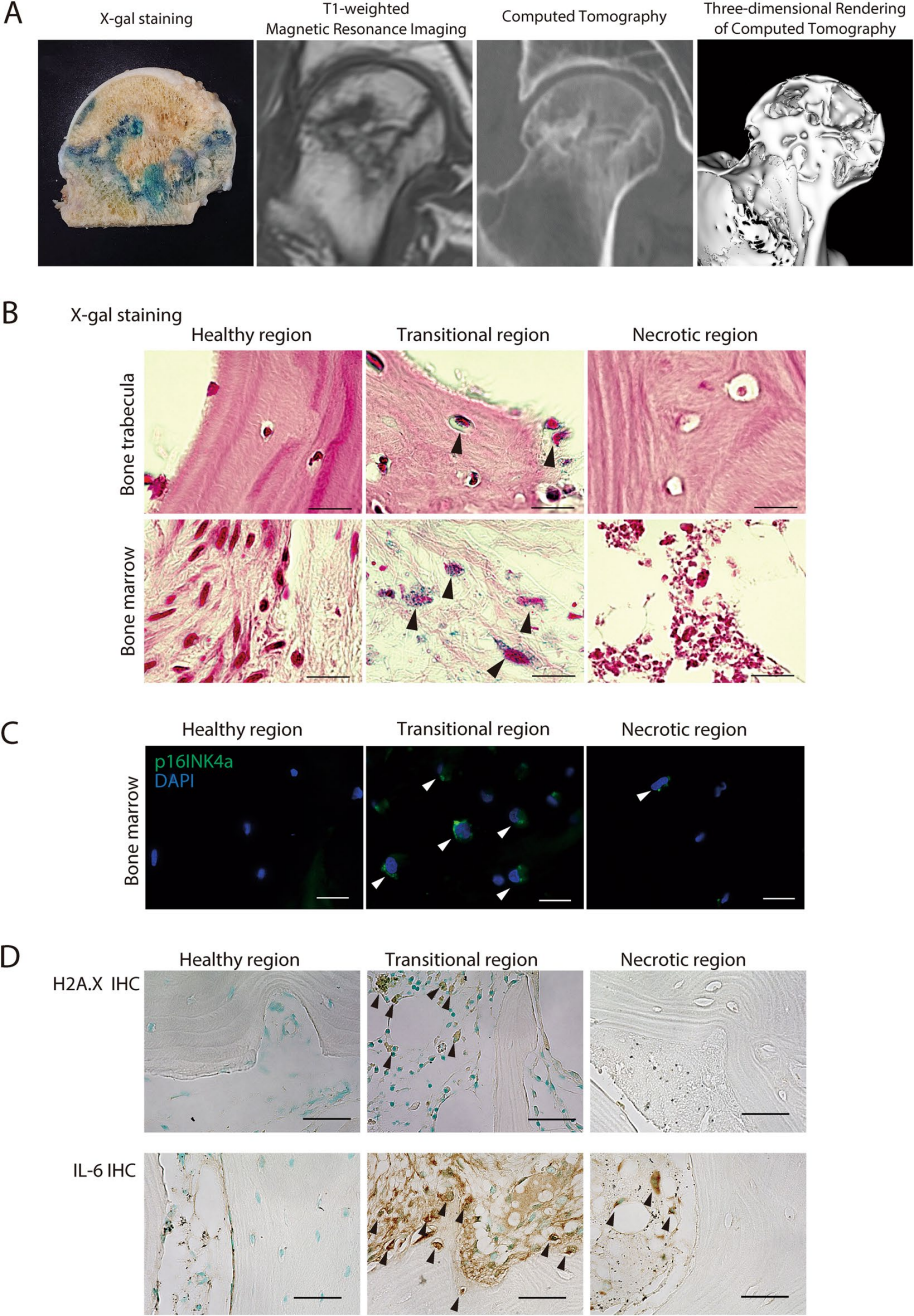
Senescent cell accumulation often occurs in the transitional region. (**A**) X-gal staining of the femoral head and MRI and CT images of nearly the same region. The band-shaped area-stained blue by X-gal was consistent with the low signal band by T1-weighted MRI and with the banded sclerotic image generated by CT, respectively. (**B**) Paraffin sections of the trabecula and bone marrow in the healthy, transitional, and necrotic regions in ONFH, X-gal stained. Cells in the trabecula of the transitional region stained blue by X-gal (black arrows) and occurred in the bone marrow. No blue staining was observed in the healthy region. Empty lacunae were seen in the necrotic region. Counterstained with nuclear fast red (NFR). Scale bar = 20 μm. (**C**) Immunofluorescence staining of p16INK4a in the healthy, transitional, and necrotic region of the femoral head in ONFH. White arrows indicate p16INK4a-positive cells. Scale bar = 20 μm. (**D**) Immunohistochemical staining for H2A.X in the healthy, transitional, and necrotic regions. Black arrows indicate H2A.X-positive cells. Scale bar = 20 μm. (**E**). Immunohistochemical staining for IL-6 in the healthy, transitional, and necrotic regions. Black arrows indicate IL-6-positive cells. Scale bar = 20 μm.

We investigated whether senescent bone cells occur in the femoral head of human ONFH. Senescence-associated β-galactosidase activity was determined by subjecting to X-gal staining the femoral heads of patients who had undergone THA for ONFH. Following approval by the ethics committee, the femoral heads of patients < 65 year old were collected to minimize the influence of individual aging. Strong X-gal staining was observed as a distal convex serpentine band inside the ONFH head. All eight assessed femoral heads showed similar band staining. In contrast, X-gal staining was weak in the necrotic region and the femoral neck (Fig. 1). X-gal staining area was detected in the transitional region of ONFH. This finding was consistent with the low-band image in T1-weighted magnetic resonance imaging (MRI) and the band sclerosis image in computed tomography (CT)^34^ (Fig. 2A). We then investigated the femoral head in osteoarthritis (OA) to confirm the specificity of X-gal staining for ONFH (Fig. 1). X-gal staining was faintly present in the articular cartilage in the femoral head of osteoarthritis. Hence, we confirmed that X-gal-positive staining is specific to ONFH (Fig. 1).

High-magnification light microscopy (Fig. 2B) revealed senescence-related granular blue staining cells in the trabecular lacunae and the bone marrow of the transitional region. By contrast, the trabeculae and cells in the bone marrow did not present with X-gal staining in the healthy region. Empty lacunae were seen in the trabeculae of the necrotic region and this histological feature is indicative of osteonecrosis. Nevertheless, X-gal staining was negative there. Empty lacunae were observed in 13.3%, 53.0%, and 95.0% of the healthy, transitional, and necrotic regions, respectively.

We also investigated the expression of the cellular senescence marker p16INK4a and phospho-histone H2A.X. Phospho-histone H2A.X serves as an index of DNA damage and a marker of cellular senescence. We found p16INK4a-positive cells in the transitional region but not in the healthy region (Fig. 2C). Whereas phospho-histone H2A.X-positive cells were observed in cells in the bone marrow of the transitional region, no positive cells were detected in the healthy region (Fig. 2D). In the transitional region, we detected high expression levels of IL-6, a marker of SASP, in numerous cells in the bone marrow and some cells in the bone trabeculae (Fig. 2D). Furthermore, immunostained IL-6 was detected in some cells within bone marrow in the necrotic region, although not in the healthy region. Senescent cells accumulated in the region surrounding the area of necrosis. The former is thought to be the zone where osteonecrosis repair occurs (Fig. 2A,B).

### Cellular senescence occurs in MSCs, osteoblasts, and osteocytes in human ONFH

**Figure 3:**
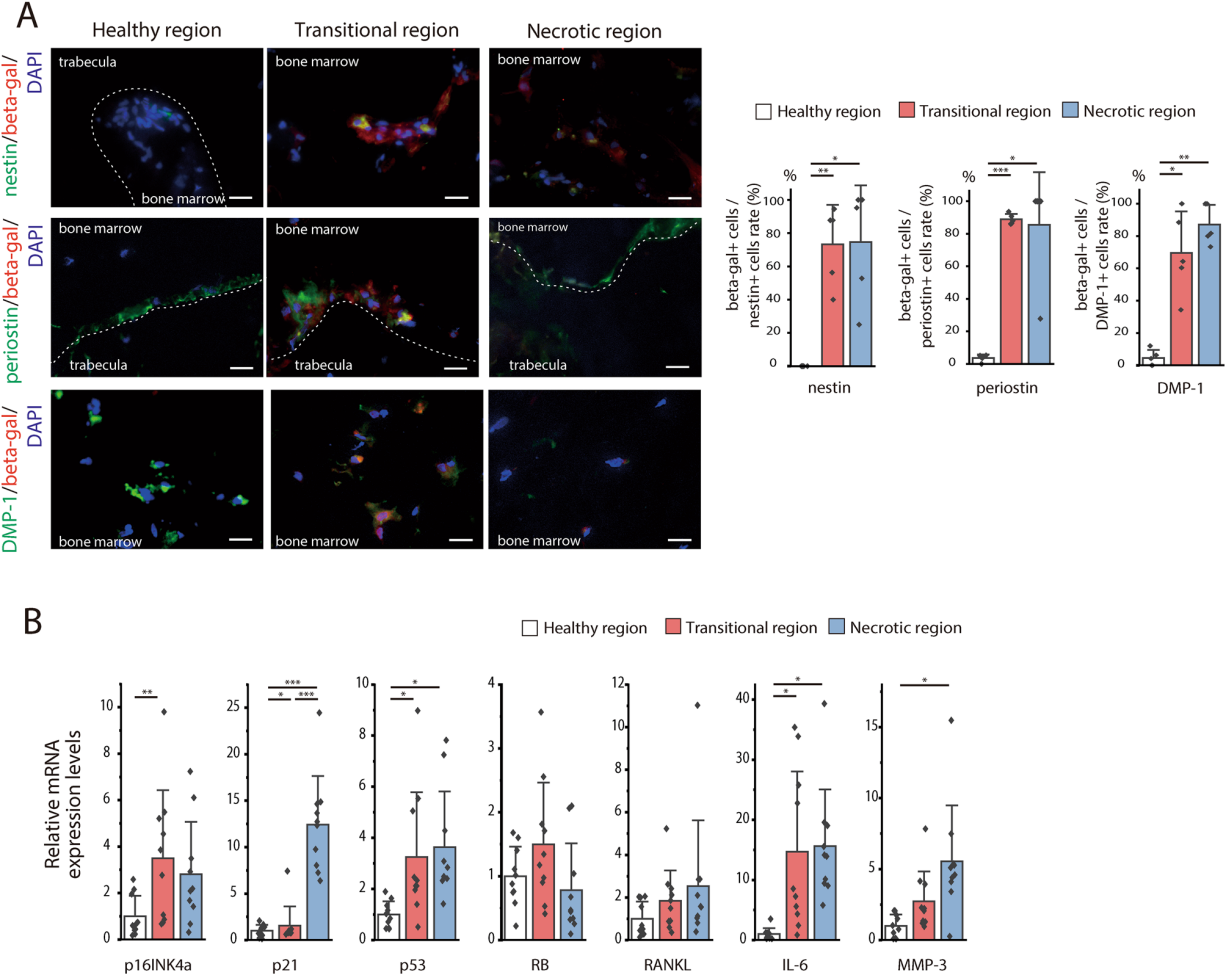
Senescence occurred in the MSCs, osteoblasts, and osteocytes in the transitional and necrotic regions of ONFH. (**A**) Beta-galactosidase immunofluorescence co-stained for nestin, periostin, and DMP-1 in the healthy, transitional, and necrotic region, respectively. Duplicated nestin, periostin, or DMP-1 signals and the senescence marker β-galactosidase were observed in the transitional and necrotic regions but not in the healthy region. Dashed lines indicate the trabecular surface. Scale bar = 20 μm. The graphs show the proportions of β-galactosidase-positive cells among the nestin-, periostin-, or DMP1-positive cells in each region (graph; n = 5). Data are shown as a dot plot and are presented as the means ± standard deviation. *p < 0.05 and **p < 0.01 by one-way, repeated-measures ANOVA with the Bonferroni post-hoc test. (**B**) RT-PCR analysis of the relative expression levels of *p16INK4a, p21, p53, RB, RANKL, IL-6,* and *MMP-3* implicated in cellular senescence in each region (n = 10). Data are shown as a dot plot and are presented as the means ± standard deviation. *p < 0.05, **p < 0.01 and ***p < 0.001 by one-way, repeated-measures ANOVA with the Bonferroni post-hoc test.

We then endeavored to determine which bone cells show cellular senescence in each region. Immunofluorescence (IF) staining revealed numerous β-galactosidase-positive cells in the transitional and necrotic regions but very few in the healthy region of ONFH (Fig. 3A).

Double staining with β-galactosidase, the MSC marker nestin, the osteoblast marker periostin, or the osteocyte marker dentin matrix acidic phosphoprotein 1 (DMP-1) was performed on the healthy, transitional, and necrotic regions (Fig. 3B). The proportions of β-galactosidase-positive cells among the nestin-positive cells in the healthy, transitional, and necrotic regions were 0.0 ± 0.0%, 73.2 ± 21.3%, and 74.6 ± 30.5%, respectively. The proportions of β-galactosidase-positive cells among the periostin-positive cells in the healthy, transitional, and necrotic regions were 3.6 ± 1.9%, 89.0 ± 2.8%, and 85.6 ± 28.9%, respectively. The proportions of β-galactosidase-positive cells among the DMP-1-positive cells in the healthy, transitional, and necrotic regions were 4.5 ± 4.5%, 69.5 ± 23.0%, and 87.0 ± 11.0%, respectively. Thus, β-galactosidase staining disclosed that cellular senescence was positive for most of the MSC, osteoblasts, and osteocytes of the transitional and necrotic regions. We also evaluated gene expression in the bone tissue of each region. Compared with the healthy region, the cellular senescence genes *p16ink4a* and *p53* and the SASP marker gene *IL-6* were upregulated in the transitional region (Fig. 3B). The *p21*, *p53* (although not *p16INK4a*), *IL-6*, and *MMP-3* genes were upregulated in the necrotic region compared to the healthy region. Hence, senescent cells were abundant in the transitional and necrotic regions of ONFH. The preceding results indicated that cellular senescence was implicated in the necrotic region where cell death occurred because of ischemia and also in the transitional region which are the reaction areas that surround the necrotic areas and undergo remodeling in ONFH. The senescent cells in these regions included MSCs, osteoblasts, and osteocytes. These mesenchymal phenotypes coordinate bone formation and homeostasis. Therefore, cellular senescence may be associated with post-necrosis bone reaction or remodeling in ONFH.

### Cellular senescence was observed in a mouse femoral ischemic osteonecrosis model

**Figure 4:**
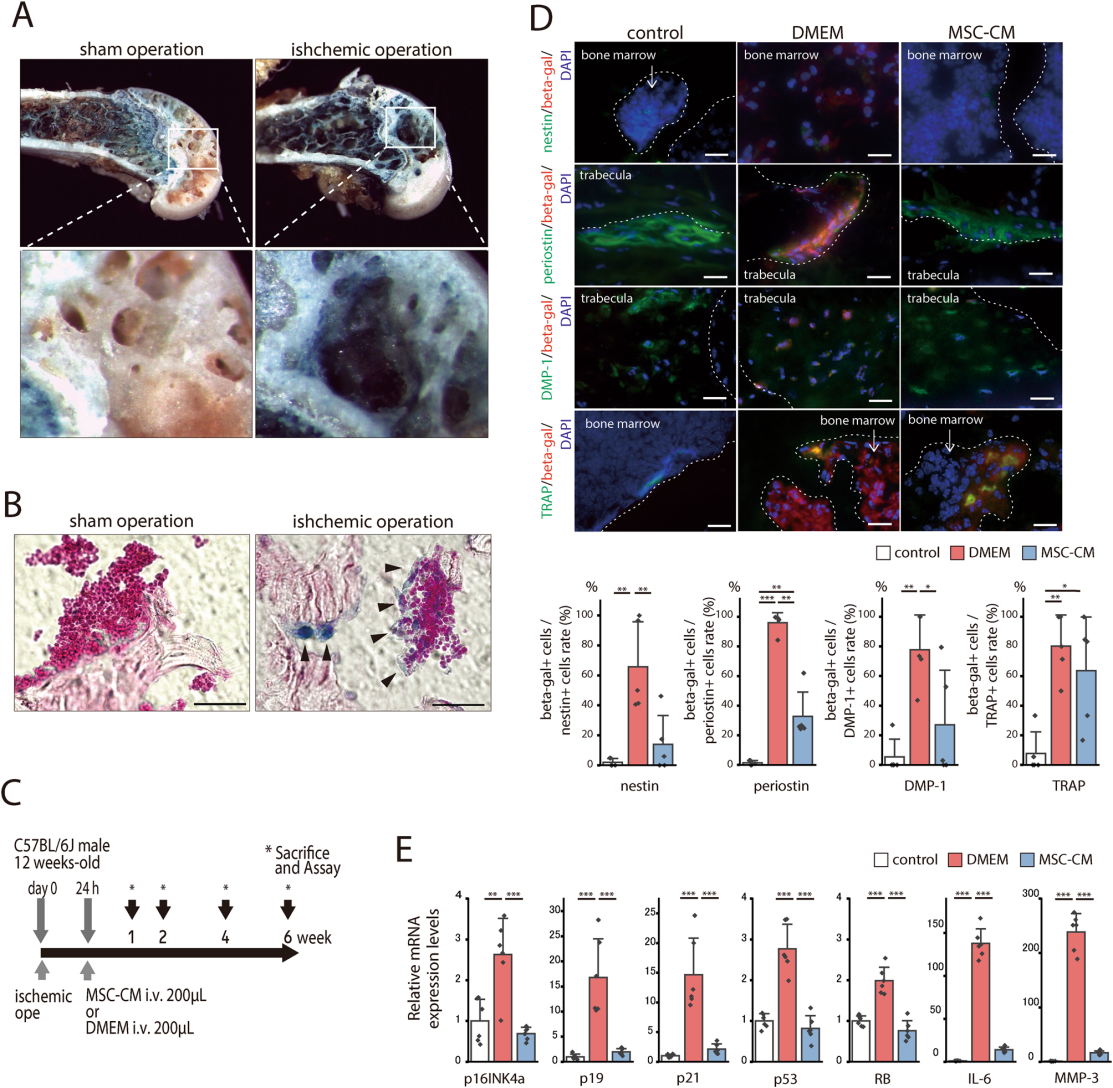
Cellular senescence was regulated by MSC-CM administration in a mouse ischemic osteonecrosis model. (**A**) Photographs of X-gal staining of mouse distal femoral epiphysis. Sham and ischemic operations were conducted and euthanasia, tissue excision, and staining were performed 1 week later. (**B**) Non-demineralized histological sections of X-gal- and NFR-stained distal femoral epiphyses are shown. Scale bar = 20 μm. Cells stained blue are indicated by black arrows and were found on the surfaces of the bone trabecular and in the bone marrow cells of mice subjected to the ischemic operation. (**C**) Development of the mouse ischemic osteonecrosis model and schedule for MSC-CM injection are shown. i.v., intravenous. (**D**) Beta-galactosidase immunofluorescence co-stained for nestin, periostin, DMP-1, and TRAP at 2 weeks after surgery. Scale bar = 20 µm. The graphs show the proportions of β-galactosidase-positive cells among the nestin-, periostin-, DMP-1, or TRAP-positive cells in each region (graph; n = 5/group). Data are shown as a dot plot and are presented as the means ± standard deviation. *p < 0.05, **p < 0.01, and ***p < 0.001 by one-way ANOVA followed by Tukey’s post-hoc test. (E) Gene expression in distal femoral epiphysis bone tissue was evaluated 1 week post-surgery. RT-PCR analysis of relative expression levels of *p16INK4a, p19, p21, p53*, and *RB* implicated in cellular senescence and those of *IL-6* and *MMP3* which are senescence-associated secretory phenotype (SASP) genes (n = 6/group). Data are shown as a dot plot and are presented as the means ± standard deviation. *p < 0.05, **p < 0.01, and ***p < 0.001 by one-way ANOVA followed by Tukey’s post-hoc test.

We examined a mouse femoral ischemic osteonecrosis model to confirm the relationship between ONFH and cellular senescence. We constructed the mouse osteonecrosis model by cauterizing the blood vessels feeding the femoral epiphysis^35^. We performed X-gal staining on the mouse tissues in the same manner as human ONFH to determine whether this model is suitable for analyzing cellular senescence in osteonecrosis. X-gal positive staining was detected in the distal femur in mouse osteonecrosis at 1 week post-surgery (Fig. 4A) and at the epiphysis of the surgically induced ischemia. Both surgically induced ischemia and sham surgery presented with blue staining in the metaphysis as the latter is nonspecifically X-gal positive^36^. High-magnification light microscopy (Fig. 4B) showed blue staining in the cells of the bone marrow and the trabecular bone at the epiphysis of surgically induced ischemia. In contrast, no positive staining was observed in the sham operation. Both the mouse ischemic osteonecrosis model and human ONFH exhibited positive X-gal staining in similar areas. In vitro, β-galactoscidase- and p16INK4a-positive cells were inhibited by the addition of MSC-CM to bone marrow cells derived from femoral heads of ONFH (Supplementary Fig. S1). For this reason, we investigated the therapeutic efficacy of MSC-CM in the mouse osteonecrosis model.

### Therapeutic effects of MSC-CM on cellular senescence in the mouse femoral ischemic osteonecrosis model

MSC-CM was intravenously injected 24 h after surgery in the mouse femoral ischemic osteonecrosis model (Fig. 4C). Immunofluorescence staining revealed senescent cells in the ischemic bone. β-galactosidase-positive cells were seen in the Dulbecco’s modified Eagle’s medium (DMEM) group but seldom in the control at 2 weeks post-surgery (Fig. 4D). For the control, the proportions of β-galactosidase-positive cells among the nestin-positive, periostin-positive, DMP-1-positive, and TRAP-positive cells were 1.9 ± 2.3%, 1.4 ± 5.9%, 5.4 ± 10.8%, and 7.8 ± 13.0%, respectively. MSC-CM administration suppressed β-galactosidase positivity in nestin-positive (DMEM vs. MSC-CM: 65.7 ± 26.8% vs. 13.9 ± 17.3%; *p* = 0.005), periostin-positive (96.1 ± 5.9% vs. 32.8 ± 14.7%; *p* = 0.001) or DMP-1-positive (77.7 ± 21.0% vs. 27.0 ± 33.0%; *p* = 0.025) cells. In contrast, MSC-CM administration did not suppress β-galactosidase positivity in TRAP-positive cells (DMEM vs. MSC-CM: 80.3 ± 18.8% vs. 63.6 ± 32.5%; *p* = 0.573). Thus, MSC-CM administration suppressed the senescent phenotype in MSCs, osteoblasts, and osteocytes which all have mesenchymal phenotypes and are senomorphic. On the other hand, MSC-CM did not inhibit cellular senescence in senescent osteoclasts.

The *p16INK4a, p19, p21, p53*, and *RB* associated with cellular senescence were upregulated in the DMEM but not the MSC-CM group (Fig. 4E). The SASP factors *IL-6* and *MMP3* were also upregulated in the DMEM but not the MSC-CM. The preceding results indicate that MSC-CM administration suppressed senescence and SASP in mice subjected to ischemic osteonecrosis.

### Cellular senescence was prolonged in ischemic osteonecrosis

**Figure 5:**
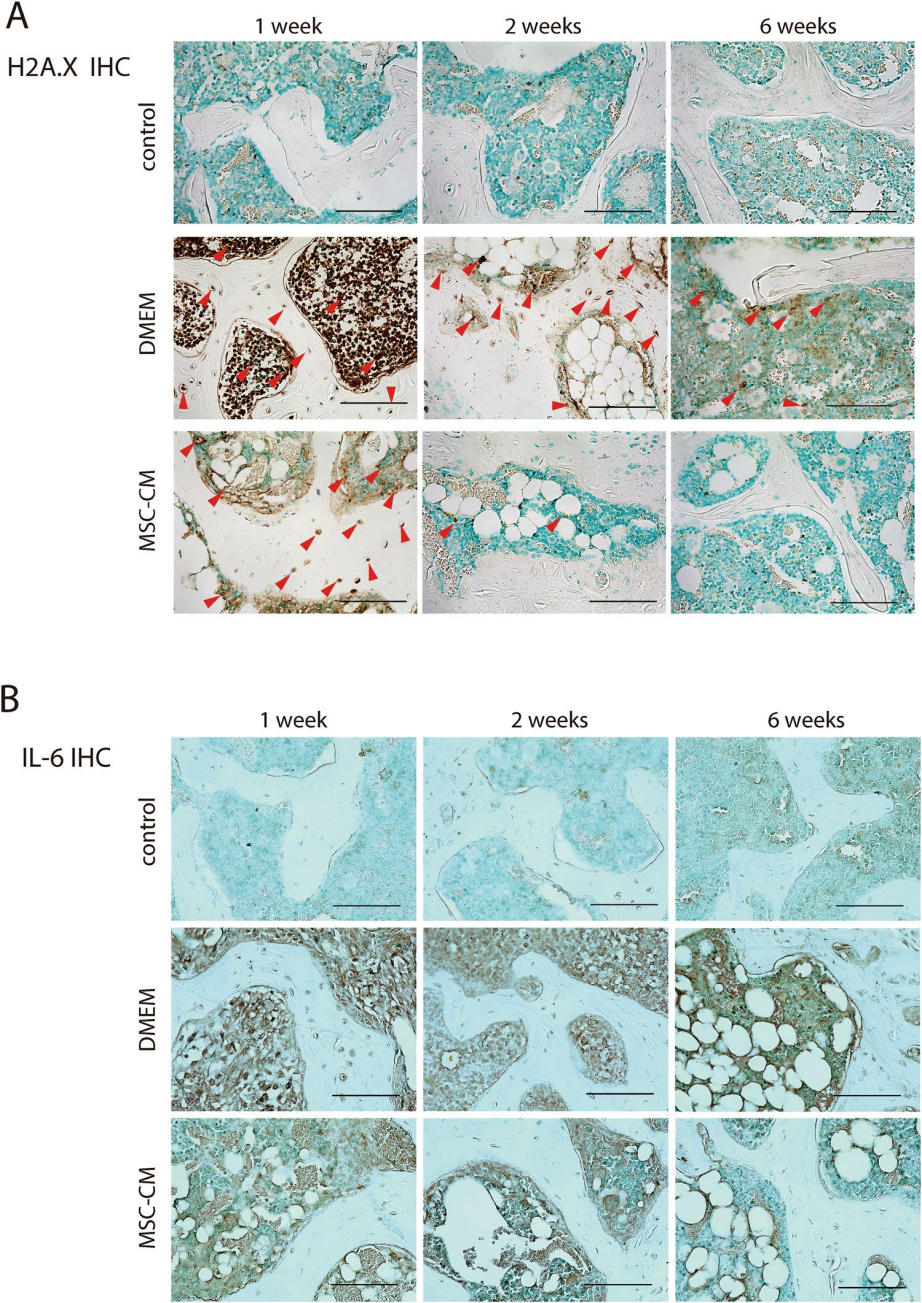
MSC-CM prevents prolongation of cellular senescence and SASP at 6 weeks post-operatively. (**A**) Immunohistochemical staining for H2A.X at 1, 2, and 6 weeks after surgery. Red arrows indicate H2A.X-positive cells. Counterstained with methyl green. Scale bar = 100 μm. (**B**). Immunohistochemical staining for IL-6 at 1, 2, and 6 weeks after surgery. Counterstained with methyl green. Scale bar = 100 μm.

IHC staining to detect the expression of H2A.X, a marker of cellular senescence. At 1 week post-operatively, we detected increases in the expression of H2A.X in both MSC-CM and DMEM, bone marrow, and bone trabeculae. In contrast to DMEM, in which H2A.X expression remained stable up to 6 weeks post-operatively, we detected a reduction in H2A.X expression over time in MSC-CM, which was similar to the control at 6 weeks post-operatively (Fig. 5A). We subsequently examined the changes in SASP over time. IL-6 was also expressed in response to the induction of ischemic osteonecrosis. At 6 weeks post-operatively, whereas we detected relatively little positive intramedullary expression in MSC-CM, high levels of residual intramedullary expression were detected in DMEM, even at 6 weeks post-operatively (Fig. 5B).

### MSC-CM administration suppressed epiphyseal bone collapse in mice subjected to ischemic osteonecrosis

**Figure 6:**
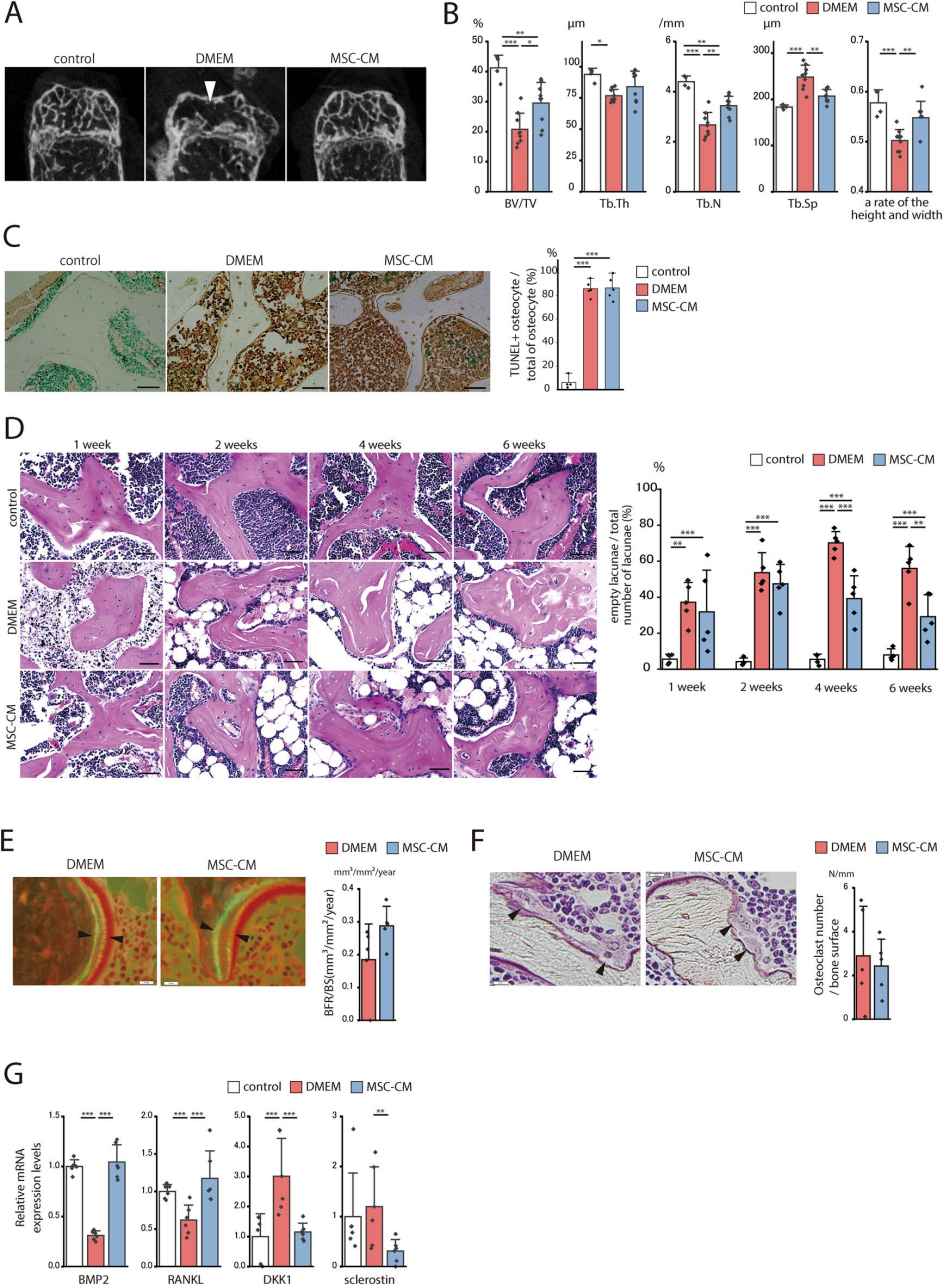
MSC-CM promotes bone formation and inhibits epiphyseal collapse. (**A**) Representative μ-CT images of coronal sections of distal femoral epiphyses from mice in the control, DMEM, and MSC-CM groups at 6 weeks post-surgery. Arrows indicate regions of decreased trabecular density. (**B**) Trabecular bone phenotype of distal femoral epiphysis was quantified by μ-CT. Trabecular bone volume/total volume (BV/TV), trabecular thickness (Tb.Th), trabecular number (Tb.N), and trabecular separation (Tb.Sp) are shown. The ratio of epiphyseal height to width is shown. Control: n = 4; DMEM: n = 9; MSC-CM: n = 9. (**C**) Representative TUNEL-stained images of formalin-fixed tissue specimens from mice in the control, DMEM, and MSC-CM groups at 1 week post-surgery. Scale bar = 20 μm. Ratios of the mean number of TUNEL-positive cells to the total number of osteocytes are shown in the graphs. Control: n = 4; DMEM: n = 5; MSC-CM: n = 5. (**D**) Osteocyte lacunae in the distal femoral epiphysis by H.E staining were evaluated between one and six weeks after surgery. Nuclei were found in the lacunae of the control group whereas empty lacunae (indicating osteocyte cell death) were observed in the DMEM and MSC-CM groups. Scale bar = 20 μm. The ratios of empty to total lacunae are shown in the graphs. Control: n = 4; DMEM: n = 5; MSC-CM: n = 5 at 1, 2, 4, and 6 weeks post-surgery. (**E**) Calcein double-labeling images of the mineralized surface at 4 weeks post-surgery. Scale bar = 10 μm. The width of the double staining area is indicated by black arrows. Bone formation rate per unit bone surface (BFR/BS, μm^3^/μm^2^/year) is shown on the graph. DMEM: n = 5; MSC-CM: n = 5. (**F**) Representative Villanueva bone staining. Black arrows indicate osteoclasts adhering to the trabecular surface. Osteoclast number/bone surface (N.Oc/Bs) is shown on the graph. DMEM: n = 5; MSC-CM: n = 5. (**G**) Gene expression in the femoral epiphysis bone tissue was evaluated at 1 week post-surgery. RT-PCR analysis of the relative expression levels of *BMP-2, RANKL, DKK1,* and *sclerostin* (n = 6/group). (**B**–**D**, **G**) Data are shown as a dot plot and are presented as the means ± standard deviation. *p < 0.05, **p < 0.01, and ***p < 0.001 by one-way ANOVA followed by Tukey’s post-hoc test. (**E**, **F**) Two-tailed unpaired *t*-test.

Epiphyseal bone collapse occurred 6 weeks after the ischemic surgery and was more evident in the DMEM than in the MSC-CM mice. Coronal images disclosed the collapse of the distal femoral epiphysis in the DMEM mice (Fig. 6A). The height/width ratio is an index of head collapse^35^ and was lower in the DMEM than in the control group (*p* < 0.001) (Fig. 6B). The head height/width ratio was also lower in the MSC-CM than the DMEM group (*p* = 0.005). MSC-CM resulted in relatively higher bone volume/tissue volume (BV/TV) (*p* = 0.014) and trabecular number (Tb.N) (*p* = 0.002) and lower trabecular separation (Tb.Sp) (*p* = 0.001). However, there was no significant difference in trabecular thickness (Tb.Th) (*p* = 0.222) between treatments. Hence, MSC-CM administration suppressed femoral head bone collapse by inhibiting epiphyseal bone loss in mice subjected to ischemic osteonecrosis.

### MSC-CM administration did not inhibit ischemia-induced osteocyte death but promoted bone formation after ischemic osteonecrosis

TUNEL staining was performed at 1 week post-surgery to determine whether MSC-CM administration inhibits cell death in bone tissue. Only a few TUNEL-positive cells were found in the lacunae (6.2 ± 4.6%) of the control (Fig. 6C). After the ischemic operation, most cells in the DMEM and MSC-CM groups were dead and there was no significant difference between them (DMEM vs. MSC-CM: 85.8 ± 6.4 vs. 86.3 ± 8.8; *p* = 0.993). Thus, MSC-CM did not inhibit cell death after the ischemic operation in the mouse osteonecrosis model.

Empty lacunae were subjected to H&E staining and examined to determine the effects of the ischemic operation and the mouse osteonecrosis model on cell death and bone formation in the bone tissue. A similar analysis was conducted by Kamiya et al.^35^. The empty lacunae increased up to 4 weeks and decreased at 6 weeks post-surgery in the DMEM group. The empty lacunae were always slightly < 10% between 1 and 6 weeks post-surgery in the control (Fig. 6D). In contrast, the empty lacunae increased up to 2 weeks and at decreased 4 weeks post-surgery in the MSC-CM group. However, there were no significant differences between the DMEM and MSC-CM groups at 1 week (DMEM vs. MSC-CM: 37.2 ± 9.9% vs. 31.9 ± 20.7%; *p* = 0.856) or 2 weeks (53.7 ± 9.9% vs. 47.4 ± 9.6%; *p* = 0.098) post-surgery. There were significantly fewer empty lacunae in the MSC-CM than in the DMEM group at 4 weeks (DMEM vs. MSC-CM: 70.2 ± 5.7% vs. 39.2 ± 11.4%; *p* < 0.001) and 6 weeks (56.0 ± 10.9% vs. 29.2 ± 10.8%; *p* = 0.005) post-surgery. In the MSC-CM group, the necrotic bone trabeculae with empty lacunae were surrounded by bone trabeculae with nucleated lacunae at 4 weeks post-surgery.

Histological observations of the mouse ischemic osteonecrosis model disclosed that new bone formation started at 4 weeks post-surgery. For the DMEM group, necrotic bone trabeculae with empty lacunae were only slightly surrounded by bone trabeculae with nucleated lacunae at 4 weeks post-surgery. For the MSC-CM group, however, necrotic bone trabeculae with empty lacunae were surrounded by bone trabeculae with nucleated lacunae at 4 weeks post-surgery. At 2 weeks post-operatively, the percentage VEGFR2-positive area in the epiphyseal medullary cavity of the MSC-CM group was higher than that in the DMEM group (Supplementary Fig. S2). This finding indicated that in response to the induction of ischemic osteonecrosis, MSC-CM induced revascularization and promoted bone formation.

Histomorphometry was performed at 4 weeks after ischemic osteonecrosis to investigate the effects of MSC-CM on the numbers of osteoblasts and osteoclasts. Comparison of the DMEM group (n = 5) and MSC-CM group (n = 5), revealed that these cells remodel bone by forming and resorbing it, respectively. Recall that H&E staining revealed relative differences in the proportions of empty lacunae at this time. The histomorphometry showed that the numbers of osteoblasts and osteoclasts did not significantly differ between the DMEM and MSC-CM groups (DMEM vs. MSC-CM: 23.0 ± 11.7/mm^2^ vs. 21.8 ± 7.0/mm^2^; *p* = 0.860; Cohen’s d = − 0.115; 2.9 ± 2.0/mm^2^ vs. 2.4 ± 1.1/mm^2^; *p* = 0.698; Cohen’s d = − 0.254).

We then used double fluorochrome labeling to assess the dynamic bone formation and measure its parameters. These factors had comparatively higher values in the MSC-CM group (Fig. 6E). The bone formation rate per bone surface (BFS/BS) was higher in the MSC-CM than in the DMEM group (MSC-CM vs. DMEM: 0.29 ± 0.03 mm^3^/mm^2^/yr vs. 0.18 ± 0.04 mm^3^/mm^2^/yr; *p* = 0.099; Cohen’s d = 1.18). However, we detected no significant difference with respect to the number of osteoclasts (Fig. 6F). MSC-CM increased the relative BMP2 and RANKL RNA expression levels which means that MSC-CM promoted bone formation. In contrast, the MSC-CM treatment decreased the relative RNA levels of DKK1 and sclerostin which inhibit bone formation (Fig. 6G). These results indicate that MSC-CM administration increases bone formation but not the number of osteoblasts.

## Discussion

To the best of our knowledge, the present study is the first to demonstrate that senescent cells accumulate in the transitional region of human ONFH. MSCs, osteoblasts, and osteocytes in the transitional region showed increased β-galactosidase activity and upregulation of the cellular senescence-related genes *p16INK4a, p21*, *p53,* and *IL-6*. Animal studies revealed that ischemic osteonecrosis increased β-galactosidase activity and H2A.X expression and upregulated cellular senescence-related and SASP genes. The administration of serum-free conditioned media from human bone marrow-derived mesenchymal stem cells did not inhibit ischemia-induced osteocyte cell death. Rather, it promoted bone formation and inhibited epiphyseal bone collapse.

The ONFH transitional region is sometimes referred to as the repair layer as it surrounds the necrotic region and forms bone^34,37,38^. The present study revealed senescent cells in the transitional region where bone formation should progress. There were more osteoblasts in the transitional region than in the healthy layer^37^. Nevertheless, the remodeling that occurred in the transitional region did not remodel the necrotic region and the latter collapsed^1^. Only a portion of the transitional region was destroyed and transformed from a healthy to a necrotic area. However, other parts of the transitional region and most senescent cells there remained. In piglets, femoral head necrosis enhanced bone formation by flushing out the cells as well as the SASP HMGB1 from the osteoclasts^39^. The preceding findings suggest that controlling the accumulation of cellular senescence is associated with bone formation.

Watanabe et al.^29^ elucidated the involvement of osteonecrosis and cellular senescence in BRONJ. Few β-galactosidase-positive cells were present in the tooth sockets after extraction. In contrast, they were present in an osteonecrosis model induced by zoledronic acid administration after tooth extraction. Furthermore, zoledronate administration upregulated *p21* and *RB* (cellular senescence-related genes), *IL-6* (interleukin-6; proinflammatory cytokine), and *MMP3* (matrix metalloprotein 3; extracellular matrix). The administration of extracellular vesicles derived from MSC-CM to the BRONJ model virtually eliminated the β-galactosidase-positive cells and downregulated *p21, RB, IL-6,* and *MMP3*. Extracellular vesicles from MSC-CM prevented senescence of the cells involved in wound healing, promoted bone formation, and prevented BRONJ. The secreting factor in MSC-CM suppresses the senescent phenotype and functions as a senomorphic. In this study, we found that MSC-CM inhibited the senescence of bone marrow cells collected from the femoral heads of ONFH. Hence, it is a senotherapeutic agent targeting cellular senescence^31,32^. MSC-CM suppressed cellular senescence in bone marrow MSC from aged mice and in fibroblasts subjected to chronic hyperglycemia^32,33^. The administration of MSC-CM instead of direct MSC transplantation would mitigate the risk of the side effects associated with stem cell-based regenerative medicine such as unwanted cellular differentiation and post-transplantation pulmonary embolism^40^.

Osteocyte senescence caused by radiation and aging results in bone loss and fragility^28,30,41–43^. Osteocytes regulate bone and mineral homeostasis by networking throughout the skeletal system^44^. To the best of our knowledge, the present study is the first to demonstrate cellular senescence in an ischemic osteonecrosis model. The induced ischemic osteonecrosis caused the death of most osteocytes in bone tissue and promoted senescence in the surviving cells. When healthy cells undergo irreparable DNA damage, they become senescent and their growth is suppressed^20^. Senescence was observed in DMP-1-positive osteocytes, nestin-positive MSCs, and periostin-positive osteoblasts. The latter can differentiate into osteocytes. However, we detected no inhibitory effect on osteoclasts. In telomerase deficiency, whereas osteoblast differentiation is impaired, osteoclast differentiation and function remain unimpaired^45,46^. Differences in the effects of cellular senescence on mesenchymal cells and osteoclasts may have contributed to differences in the inhibitory effects of MSC-CM on cellular senescence. SASP secretion may create an unfavorable proinflammatory microenvironment. Here, we confirmed IL-6 and MMP3 upregulation in SASP. Therefore, this mechanism may contribute to the inflammation and bone fragility characteristic of ischemic osteonecrosis. Cellular senescence and SASP attributable to the induction of ischemic osteonecrosis persisted for 6 weeks after surgery. These findings indicate that senescent cells remained and continued to affect tissues over the long term. Indeed, in the femoral heads of human ONFH, we detected the expression of H2A.X and IL-6, regardless of whether the period of arthropathic changes had passed prior to surgery. Induction of bone fragility resulted in epiphyseal collapse and the loss of bone volume. However, bone fragility can be improved by suppressing osteocyte senescence^41,42^. The suppression of cellular senescence by administration of the senomorphic agent MSC-CM maintained bone volume and prevented epiphyseal collapse. The bioactive factors secreted by MSC inhibited apoptosis in damaged tissues^47,48^. MSC-CM was ineffectual at inhibiting cell death after ischemia induction. By contrast, MSC-CM administration halted the senescence of mesenchymal phenotypes such as MSCs, osteoblasts, and osteocytes and maintained them in a state similar to that of the control group (Supplementary Fig. S3). Furthermore, MSC-CM administration downregulated IL6 and MMP3 and blocked the transmission of chronic proinflammatory signals that might have impeded bone formation. The early decrease in empty lacunae, expression of VEGFR2, and the trend toward accelerating bone formation suggest that MSC-CM might initiate osteogenesis after the induction of ischemic osteonecrosis. In this manner, MSC-CM maintains bone volume and suppresses the epiphyseal collapse. The in vivo experiments showed that the suppression of cellular senescence mitigated bone collapse even though cell death could not be prevented after ischemia induction.

Invasive surgical therapy can be avoided and normal pain-free hip joint motor function can be maintained if post-osteonecrosis femoral head collapse can be prevented or mitigated in patients with ONFH^49^. The foregoing findings indicate that cellular senescence is a potential therapeutic target for ischemic osteonecrosis. The clinical use of MSC-CM involves the need for MSC culture and thus entail a potential risk of infection. To avoid the necessity of using MSCs, in future research, it will be important to identify the factors in MSC-CM that suppresses cellular senescence, such as growth factors, extracellular vesicles, and miRNAs. In the future, not only the form of senomorphic, but also the effect of senolysis, which directly destroys senescent cells, should be clarified.

The present study was associated with several limitations. First, the human bone samples used were restricted to cases of osteonecrosis followed by severe crushing or arthropathic changes. Patients who are in the early stages of the disease shortly after the onset of necrosis and present with no epiphyseal collapse should not undergo total hip arthroplasty (THA) and should be treated by joint preservation. For these reasons, it was impossible to collect early-stage human samples. However, radiological features characteristic of osteosclerosis in the transitional region were observed in the early stages of the disease^38^. Hence, it is important to examine the transitional region at all disease stages. Second, we only used a mouse distal femoral osteonecrosis model as no reliable mouse femoral osteonecrosis model is currently available. Osteogenesis (direct calcified cartilage ossification) of the femoral head differs between mice and humans as the latter have a secondary ossification center^50^. In mice, only the distal femoral epiphysis undergoes secondary ossification. For this reason, it is used to study ischemic osteonecrosis. This result of the present study should be confirmed in a large animal model in the next phase. This is because porcine femoral heads have secondary ossification centers and have similar histological features to humans^51^.

## Conclusions

In the present work, we observed cellular senescence in the femoral heads of ONFH patients. Senescent cells were detected in both the necrotic region of ONFH and the transitional region where bone formation should have progressed. MSC-CM did not suppress ischemia-induced cell death. Nevertheless, it prevented cellular senescence in the bone tissue, promoted bone formation, and prevented epiphyseal collapse. Therefore, the regulation of cellular senescence may be a therapeutic target for ONFH.

## Materials and methods

### Human

Femoral head samples were evaluated to determine how cellular senescence is implicated in human ONFH. ONFH and OA tissues were obtained from THA surgeries. All studies involving humans were performed in accordance with the protocol of the Institutional Review Board of Nagoya University Hospital (No. 2021-0279) and approved by the Ethical Review Committee of the Nagoya University Graduate School of Medicine. Informed consent was obtained from the patients with OA or ONFH who participated in the present study. All samples were anonymized before use. The exclusion criteria were prior surgeries, traumatic osteonecrosis of the femoral head, and age > 65 year. Older patients were omitted to eliminate the confounding effect of individual aging. Patients were not burdened with an addendum because surgically excised femoral heads are usually discarded. The mean age of the 12 cases of ONFH was 50.2 ± 11.5 year, the age range was 27–64 year, and there were three females and nine males. There were four cases of Association Research Circulation Osseous (ARCO) stage 3A, six cases of ARCO stage 3B, and four cases of ARCO stage 4^52^. There were two patients (one male and one female) with OA and they were in their 60 s. All methods were carried out in accordance with relevant guidelines and regulations.

### Animals

All mouse experiment protocols were approved by the Animal Care and Use Committee of the Nagoya University Graduate School of Medicine and performed in accordance with all relevant guidelines (No. 220396-004). The current study was performed according to ARRIVE guidelines and all experimental procedures were conducted in accordance with international guidelines for the care and use of laboratory animals. The mice were housed under a 12 h light–dark cycle and given free access to feed (a standard commercial diet) and water. C57BL/6J were obtained from SLC Japan Inc. (Tokyo, Japan). C57BL/6J mice aged 12 weeks were used^53^. The mouse model of ischemic osteonecrosis was constructed as previously described^35^. Briefly, the mice were anesthetized with isoflurane and to induce ischemic osteonecrosis, the popliteal, medial, central, and lateral genicular blood vessels supplying the right distal femoral epiphysis were cauterized under a SZ40 stereomicroscope at 10× magnification (Olympus, Tokyo, Japan). The same blood vessels of the contralateral left distal femoral epiphysis were simply visualized under a microscope in a sham operation. The mice subjected to the sham operation served as the control.

The mice were randomly assigned to the control, DMEM, or MSC-CM group to evaluate the effects of MSC-CM. The mice in the control group underwent a sham operation on the right knee as previously described. The mice in the DMEM and MSC-CM groups underwent an ischemic operation on the right knee as previously described. The mice in the control and DMEM groups were infused with 200 μL DMEM in the tail vein the day after the surgery. The mice in the MSC-CM group were infused with 200 μL MSC-CM in the tail vein the day after the surgery. Four, five, and five mice in the control, DMEM, and MSC-CM groups, respectively, were sacrificed 1 week after surgery. Nine, ten, and ten mice in the control, DMEM, and MSC-CM groups, respectively, were sacrificed 2 weeks after surgery. Four, five, and five mice in the control, DMEM, and MSC-CM groups, respectively, were sacrificed 4 weeks after surgery. Four, nine, and nine mice in the control, DMEM, and MSC-CM groups, respectively, were sacrificed 6 weeks after surgery.

## MSC-CM preparation

Human MSCs were acquired from Lonza, Inc. (Walkersville, MD, USA) and cultured at 37 °C under a 5% CO_2_/95% air (/v) atmosphere in MSC basal medium (Lonza, Inc.) containing MSC-GM Single Quots (Lonza, Inc.). The hMSCs at the five-passage stage were used. At 80% confluence, the cells were washed three times with phosphate-buffered saline (PBS) and replenished with serum-free DMEM (Sigma-Aldrich Corp., Tokyo, Japan) containing a 1% antibiotic–antimycotic solution (Sigma-Aldrich Corp., Tokyo, Japan). The cell-cultured, conditioned media were collected after 48 h incubation and centrifuged at 440×*g* and 4 °C for 5 min. The supernatants were centrifuged at 17,400×*g* and 4 °C for 3 min to isolate the MSC-CM. The latter were prepared immediately prior to each use. The DMEM administered to the mice was serum-free and contained the 1% antibiotic–antimycotic solution.

### Histology

Femoral heads of humans were sliced to a width of 5 mm in the coronal plane at the median level in the axial plane. All samples were stained within 6 h of being excised. Senescence-associated β-galactosidase (β-gal) activity was measured in the femoral head tissues as previously described^23^. The samples were incubated at 37 °C for 24 h in β-gal staining solutions containing 1 mg/mL 5-bromo-4-chloro-3-indolyl-β-D-galactopyranoside (X-gal), 5 mM potassium ferrocyanide, 5 mM potassium ferricyanide, 150 mM NaCl, 2 mM MgCl_2_, 0.01% (w/v) sodium deoxycholate, and 0.02% (w/v) Nonidet P-40. The stained femoral heads were then photographed. Samples were fixed in 4% (v/v) paraformaldehyde (PFA), decalcified with 10% (w/v) EDTA (Sigma-Aldrich Corp., Tokyo, Japan) at 4 °C for 3 weeks, embedded in paraffin, sectioned, and counterstained with Nuclear Fast Red (NFR).

Sham-operated and ischemia-operated mice were sacrificed 1 week after surgery. The distal femoral metaphysis and epiphysis were cut in half along the sagittal plane. The samples were subjected to β-gal staining as previously described. One-half of each sample was photographed at 4× magnification under a Leica M60 stereomicroscope (Leica, Wetzlar, Germany), whereas the remaining half was cut into 5-μm sections without decalcification, frozen using a cryostat (CM3050S-IV; Leica Microsystems, Wetzlar, Germany), counterstained with NFR, and photographed at 40× magnification under a BZ-X710 microscope (Keyence, Osaka, Japan).

### Immunohistochemistry

Decalcified paraffin slides of the femoral head of ONFH and distal femoral epiphyses from mice at 1, 2, and 6 weeks after surgery were processed for immunohistochemistry. As primary antibodies, we used rabbit anti-phospho-histone H2A.X antibody (2577; Cell Signaling Technology, Danvers, MA, USA) and IL-6 (ab6672; Abcam, Cambridge, MA), and a goat anti-rabbit IgG antibody (Nichirei Bioscience, Tokyo, Japan) was used as the secondary antibody. Staining was performed using 3,3′-diaminobenzidine tetrahydrochloride (Nichirei Bioscience, Tokyo, Japan) for 10 min, followed by counterstaining with methyl green. Stained sections were observed and photographed at 20× or 40× magnification using a BZ-X710 microscope (Keyence, Osaka, Japan).

### Immunofluorescence

A 5-mm slice of bone adjacent to the bone head section subjected to X-gal staining was decalcified with 10% (w/v) EDTA at 4 °C for 3 weeks, embedded in paraffin, sectioned, and subjected to fluorescence IF staining with p16INK4a (ab211542; Abcam, Cambridge, UK). Multiple rounds of staining were performed to disclose cellular senescence.

The necrotic, transitional, and healthy regions of the ONFH femoral head were cut into 1-cm blocks and prepared separately from the demineralized samples. Non-demineralized and unfixed 6-mm bone tissue sections were frozen and subsequently prepared using the adhesive film method developed by Kawamoto^54^. Briefly, the bone tissue was initially freeze-embedded in a water-soluble medium, following which an adhesive film was applied to the cut surface to support the frozen sample whilst being cut using a disposable tungsten carbide blade. Sections of 6-µm thickness were prepared using a cryostat (CM3050S-IV; Leica Microsystems, Wetzlar, Germany). Immunofluorescence staining was conducted with β-galactosidase (ab9361; Abcam), nestin (ab92391; Abcam), periostin (ab14041; Abcam), and DMP-1 (NBP1-45525; Novus Biologicals, Centennial, CO, USA). The sections were mounted with VectaShield mounting medium containing 4′,6-diamidino-2-phenylindole (DAPI). In vitro, Immunofluorescence staining was performed using β-galactosidase (ab9361; Abcam) and p16INK4a (ab211542; Abcam) and the sections were mounted with VectaShield mounting medium containing DAPI. The sections were examined at 40× magnification under a BZ-X710 fluorescence microscope (Keyence, Osaka, Japan) and the β-gal-positive cells were counted using a BZ-X 800 Analyzer (Keyence, Osaka, Japan). 315, 809, and 216 nestin + cells were observed in the healthy, transitional, and necrotic regions, respectively, as were 616, 2218, and 291 periostin + cells, and 630, 1321, and 321 DMP-1 + cells, respectively.

Multiplex staining for β-gal, nestin, periostin, and DMP-1 was performed on non-demineralized, unfixed mouse knee sections at 2 weeks after surgery, as previously described for human samples. The mouse knee sections were also subjected to β-galactosidase and TRAP staining (ab191496; Abcam). The cells were blindly counted using a BZX800 analyzer (Keyence, Osaka, Japan).

### Reverse transcription-quantitative polymerase chain reaction (RT-qPCR) analysis

In humans, the expression levels of *p16INK4a, p21, p53, RB, p53, RANKL, IL-6*, and *MMP-3* were determined by using RT-qPCR to measure their mRNA levels. Bone tissue from the necrotic, transitional, and healthy regions were cut into 5-mm blocks, flash-frozen immediately after excision, and pulverized with a Multi-Beads Shocker (Yasui Kikai Co. Ltd., Osaka, Japan). In mice, the expression levels of *p16INK4a, p19, p21, p53, RB, IL-6, MMP-3, BMP2, RANKL, DKK1,* and *sclerostin* were determined by measuring their mRNA levels using RT-qPCR. At 1 week post-surgery, mice were sacrificed and their distal femoral epiphyses were immediately excised. The cartilage was dissected with a scalpel and frozen. Total RNA was extracted and isolated from all bone samples with the RNeasy Mini Kit (Qiagen, Hilden, Germany) according to the manufacturer’s protocol. The relative mRNA levels of the target genes were normalized to that of glyceraldehyde 3-phosphate dehydrogenase (GAPDH). All measurements were analyzed by the 2^−ΔΔC(t)^ method^55^. The primer sequences are listed in Table S1.

### Micro-CT

Radiographic analysis of the distal femoral epiphysis was performed by subjecting the mice to µ-CT scan (Skyscan 1176; Bruker, Kontich, Belgium). The operating parameters were 50 kV X-ray voltage, 500 μA X-ray current, 0.5 mm Al filter, 0.5° rotation step, 9 μm pixel size, and no frame averaging. The µ-CT scan images were reconstructed with Skyscan NRecon software and analyzed by using its 3D algorithms according to the manufacturer’s instructions. To evaluate the degree of epiphyseal collapse, the epiphyseal height-to-width ratios were calculated using the measurement obtained for the coronal sections of the µ-CT images^35^. The region of interest was the cancellous bone at the distal femoral epiphysis surrounded by outlined cortical bone. The bone volume/tissue volume (BV/TV), trabecular thickness (Tb.Th), trabecular number (Tb.N), and trabecular separation (Tb.Sp) were calculated to delineate and define the epiphyseal trabecular compartments.

### Histological analysis

Bone specimens were fixed in 4% (v/v) PFA at 20 ℃ for 2 weeks, decalcified with 10% (w/v) EDTA (Sigma-Aldrich Corp.) at 4 °C for 3 weeks, and embedded in paraffin. Sagittal sections (5-µm) were cut using a Sakura IVS-400 sledge microtome (Sakura Seiki, Tokyo, Japan), subjected to hematoxylin and eosin (H&E) staining, and examined microscopically. One week after surgery, certain bone sections were subjected to TdT-mediated dUTP nick end labeling (TUNEL) staining (Fujifilm, Osaka, Japan) and counterstained with methyl green.

### Histomorphometry

Double labeling was performed by subcutaneously injecting the mice with 10 mg/kg calcein at 6 d and 2 d before euthanasia. Five mice in the DMEM group and five mice in the MSC-CM group underwent bone histomorphometry 4 weeks after surgery. After sacrifice, the right knee joints of mice were resected, fixed in 70% (v/v) alcohol, stained with Villanueva bone stain, and embedded in methyl methacrylate resin (Wako Pure Chemical Industry, Osaka, Japan) without decalcification. The resulting blocks were sectioned using a Reichert-Jung microtome (model 2050; Finetech Scientific Instruments, Tokyo, Japan) along the frontal plane at 5-μm thickness. An observer from an external institution blinded to the experimental treatments and groups evaluated the number of osteoblasts per bone surface (N.Ob/BS), the number of osteoclasts per bone surface (N.Oc/BS), and the bone formation rate per bone surface (BFR/BS).

### Statistical analysis

Unpaired Student’s *t*-test, one-way analysis of variance (ANOVA) followed by Tukey’s post-hoc test, and one-way repeated-measures ANOVA with Bonferroni’s post-hoc test were performed in SPSS v. 28 (IBM Corp., Armonk, NY, USA) and Origin 2023 (OriginLab Corp., Northampton, Massachusetts, USA). Effect size was determined by Cohen’s d method. Data are means ± standard deviation (SD). *p* < 0.05 was considered statistically significant.

### Other materials and methods

Details of other materials and methods are available in the Supplementary Information. This section includes detailed descriptions of isolation of human osteonecrosis of the femoral head (ONFH) cells, staining of the bone marrow of the mice, and real-time quantitative polymerase chain reaction (qPCR).

### Supplementary Information

Supplementary Information.

## Data Availability

Data will be made available upon reasonable request of the corresponding author.

## References

[1] Mont MA, Zywiel MG, Marker DR, McGrath MS, Delanois RE (2010). The natural history of untreated asymptomatic osteonecrosis of the femoral head: A systematic literature review. J. Bone Jt. Surg. Am..

[2] Kang JS (2009). Prevalence of osteonecrosis of the femoral head: A nationwide epidemiologic analysis in Korea. J. Arthroplasty..

[3] Zhao DW (2015). Prevalence of nontraumatic osteonecrosis of the femoral head and its associated risk factors in the Chinese population: Results from a nationally representative survey. Chin. Med. J. (Engl.).

[4] Mont MA, Hungerford DS (1995). Non-traumatic avascular necrosis of the femoral head. J. Bone Jt. Surg. Am..

[5] Mont MA, Jones LC, Hungerford DS (2006). Nontraumatic osteonecrosis of the femoral head: Ten years later. J. Bone Jt. Surg. Am..

[6] Talamo G (2005). Avascular necrosis of femoral and/or humeral heads in multiple myeloma: Results of a prospective study of patients treated with dexamethasone-based regimens and high-dose chemotherapy. J. Clin. Oncol..

[7] Matsuo K, Hirohata T, Sugioka Y, Ikeda M, Fukuda A (1988). Influence of alcohol intake, cigarette smoking, and occupational status on idiopathic osteonecrosis of the femoral head. Clin. Orthop. Relat. Res..

[8] Deng Z, Ren Y, Park MS, Kim HKW (2022). Damage associated molecular patterns in necrotic femoral head inhibit osteogenesis and promote fibrogenesis of mesenchymal stem cells. Bone.

[9] Zalavras C (2000). Potential aetiological factors concerning the development of osteonecrosis of the femoral head. Eur. J. Clin. Invest..

[10] Ichiseki T (2017). Involvement of necroptosis, a newly recognized cell death type, in steroid-induced osteonecrosis in a rabbit model. Int. J. Med. Sci..

[11] O'Brien CA (2004). Glucocorticoids act directly on osteoblasts and osteocytes to induce their apoptosis and reduce bone formation and strength. Endocrinology.

[12] Iuchi T (2003). Glucocorticoid excess induces superoxide production in vascular endothelial cells and elicits vascular endothelial dysfunction. Circ. Res..

[13] Kubo Y (2021). Adverse effects of oxidative stress on bone and vasculature in corticosteroid-associated osteonecrosis: potential role of nuclear factor erythroid 2-related factor 2 in cytoprotection. Antioxid. Redox Signal.

[14] Orrenius S, Gogvadze V, Zhivotovsky B (2007). Mitochondrial oxidative stress: implications for cell death. Annu. Rev. Pharmacol. Toxicol..

[15] Urbaniak JR, Coogan PG, Gunneson EB, Nunley JA (1995). Treatment of osteonecrosis of the femoral head with free vascularized fibular grafting. A long-term follow-up study of one hundred and three hips. J. Bone Jt. Surg. Am..

[16] Bozic KJ, Zurakowski D, Thornhill TS (1999). Survivorship analysis of hips treated with core decompression for nontraumatic osteonecrosis of the femoral head. J. Bone Jt. Surg. Am..

[17] Sakano S, Hasegawa Y, Torii Y, Kawasaki M, Ishiguro N (2004). Curved intertrochanteric varus osteotomy for osteonecrosis of the femoral head. J. Bone Jt. Surg. Br..

[18] Tsukanaka M (2016). Implant survival and radiographic outcome of total hip replacement in patients less than 20 years old. Acta Orthop..

[19] Mohaddes M (2019). Implant survival and patient-reported outcome following total hip arthroplasty in patients 30 years or younger: A matched cohort study of 1,008 patients in the Swedish Hip Arthroplasty Register. Acta Orthop..

[20] Campisi J, d’Adda di Fagagna F (2007). Cellular senescence: When bad things happen to good cells. Nat. Rev. Mol. Cell Biol..

[21] Coppé JP, Desprez PY, Krtolica A, Campisi J (2010). The senescence-associated secretory phenotype: The dark side of tumor suppression. Annu. Rev. Pathol..

[22] Rodier F, Campisi J (2011). Four faces of cellular senescence. J. Cell Biol..

[23] Minamino T (2002). Endothelial cell senescence in human atherosclerosis: Role of telomere in endothelial dysfunction. Circulation.

[24] Aguayo-Mazzucato C (2019). Acceleration of β cell aging determines diabetes and senolysis improves disease outcomes. Cell Metab..

[25] Desdín-Micó G (2020). T cells with dysfunctional mitochondria induce multimorbidity and premature senescence. Science.

[26] Baker DJ (2011). Clearance of p16Ink4a-positive senescent cells delays ageing-associated disorders. Nature.

[27] McCulloch K, Litherland GJ, Rai TS (2017). Cellular senescence in osteoarthritis pathology. Aging Cell.

[28] Farr JN (2016). Identification of senescent cells in the bone microenvironment. J. Bone Miner. Res..

[29] Watanabe J (2020). Extracellular vesicles of stem cells to prevent BRONJ. J. Dent. Res..

[30] Kim HN (2020). Osteocyte RANKL is required for cortical bone loss with age and is induced by senescence. JCI Insight.

[31] Di Micco R, Krizhanovsky V, Baker D, d’Adda di Fagagna F (2021). Cellular senescence in ageing: From mechanisms to therapeutic opportunities. Nat. Rev. Mol. Cell Biol..

[32] Dorronsoro A (2021). Mesenchymal stem cell-derived extracellular vesicles reduce senescence and extend health span in mouse models of aging. Aging Cell.

[33] Li M (2017). Umbilical cord-derived mesenchymal stromal cell-conditioned medium exerts in vitro antiaging effects in human fibroblasts. Cytotherapy.

[34] Murphey MD (2014). From the radiologic pathology archives imaging of osteonecrosis: Radiologic-pathologic correlation. Radiographics.

[35] Kamiya N, Yamaguchi R, Aruwajoye O, Adapala NS, Kim HK (2015). Development of a mouse model of ischemic osteonecrosis. Clin. Orthop. Relat. Res..

[36] Kopp HG, Hooper AT, Shmelkov SV, Rafii S (2007). Beta-galactosidase staining on bone marrow. The osteoclast pitfall. Histol. Histopathol..

[37] Wang C (2014). Bone microstructure and regional distribution of osteoblast and osteoclast activity in the osteonecrotic femoral head. PLoS ONE.

[38] Plenk H (2001). Magnetic resonance imaging and histology of repair in femoral head osteonecrosis. Clin. Orthop. Relat. Res..

[39] Kim HKW (2021). Minimally invasive necrotic bone washing improves bone healing after femoral head ischemic osteonecrosis: An experimental investigation in immature pigs. J. Bone Jt. Surg. Am..

[40] Herberts CA, Kwa MS, Hermsen HP (2011). Risk factors in the development of stem cell therapy. J. Transl. Med..

[41] Chandra A (2020). Targeted reduction of senescent cell burden alleviates focal radiotherapy-related bone loss. J. Bone Miner. Res..

[42] Farr JN (2017). Targeting cellular senescence prevents age-related bone loss in mice. Nat. Med..

[43] Yao Z (2020). Therapy-induced senescence drives bone loss. Cancer Res..

[44] Dallas SL, Prideaux M, Bonewald LF (2013). The osteocyte: An endocrine cell … and more. Endocr. Rev..

[45] Pignolo RJ (2008). Defects in telomere maintenance molecules impair osteoblast differentiation and promote osteoporosis. Aging Cell.

[46] Wang H (2012). Impairment of osteoblast differentiation due to proliferation-independent telomere dysfunction in mouse models of accelerated aging. Aging Cell.

[47] Caplan AI, Dennis JE (2006). Mesenchymal stem cells as trophic mediators. J. Cell. Biochem..

[48] Sakai K (2012). Human dental pulp-derived stem cells promote locomotor recovery after complete transection of the rat spinal cord by multiple neuro-regenerative mechanisms. J. Clin. Invest..

[49] Osawa Y (2022). Hip function in patients undergoing conservative treatment for osteonecrosis of the femoral head. Int. Orthop..

[50] Cole HA (2013). Differential development of the distal and proximal femoral epiphysis and physis in mice. Bone.

[51] Kim HK, Su PH, Qiu YS (2001). Histopathologic changes in growth-plate cartilage following ischemic necrosis of the capital femoral epiphysis. An experimental investigation in immature pigs. J. Bone Jt. Surg. Am..

[52] Yoon BH (2020). The 2019 revised version of association research circulation osseous staging system of osteonecrosis of the femoral head. J. Arthroplasty.

[53] Yamaguchi R, Kamiya N, Kuroyanagi G, Ren Y, Kim HKW (2021). Development of a murine model of ischemic osteonecrosis to study the effects of aging on bone repair. J. Orthop. Res..

[54] Kawamoto T (2003). Use of a new adhesive film for the preparation of multi-purpose fresh-frozen sections from hard tissues, whole-animals, insects and plants. Arch. Histol. Cytol..

[55] Livak KJ, Schmittgen TD (2001). Analysis of relative gene expression data using real-time quantitative PCR and the 2(-Delta Delta C(T)) Method. Methods.

