# Supplementary material for: Cellular senescence is associated with osteonecrosis of the femoral head while mesenchymal stem cell conditioned medium inhibits bone collapse
Source: Sci Rep. 2024 Feb 9;14:3329. doi: 10.1038/s41598-024-53400-w (PMC10858285; doi:10.1038/s41598-024-53400-w)
Supplement: Supplementary file 1 — Supplementary Information. [file 41598_2024_53400_MOESM1_ESM.docx]

**Supplementary date:**


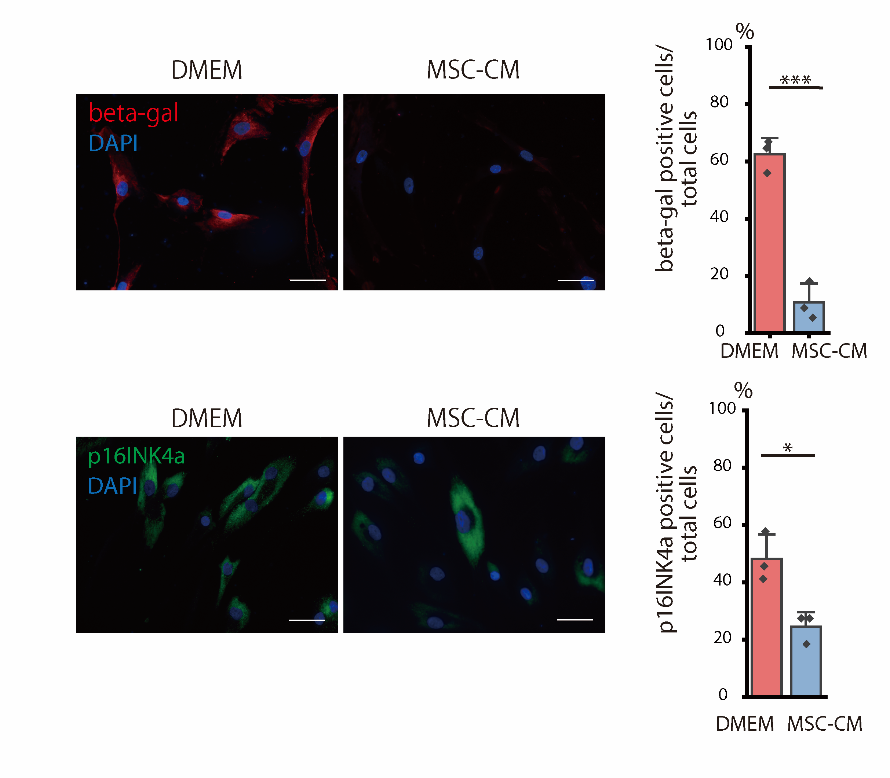


**Supplementary Figure 1. Effects of MSC-CM on MSC senescence in ONFH.** Bone marrow cells were collected from the bone trabecular fragments of the ONFH transitional region and cultured. Cells at the third passage were used in the assay. MSC-CM or PBS was added to the cultured cells to determine whether MSC-CM suppresses cellular senescence. Βeta-galactosidase-positive and p16INK4a-positive cells were observed by fluorescent immunostaining. Βeta-galactosidase-positive cells (DMEM vs. MSC-CM: 62.4 ± 4.7% vs. 10.8 ± 5.4%; p < 0.001) and p16INK4a-positive cells (48.2 ± 7.0% vs. 24.4 ± 4.2%; p = 0.022) were significantly reduced in response to MSC-CM compared with DMEM. The proportions of β-galactosidase-positive and p16INK4a-positive cells are shown in the graph. Scale bar = 50 µm. Data consists of a dot plot and the mean ± standard deviation. *P < 0.05 and ***P < 0.001 by unpaired, two-tailed Student’s *t*-test (n = 3/group).


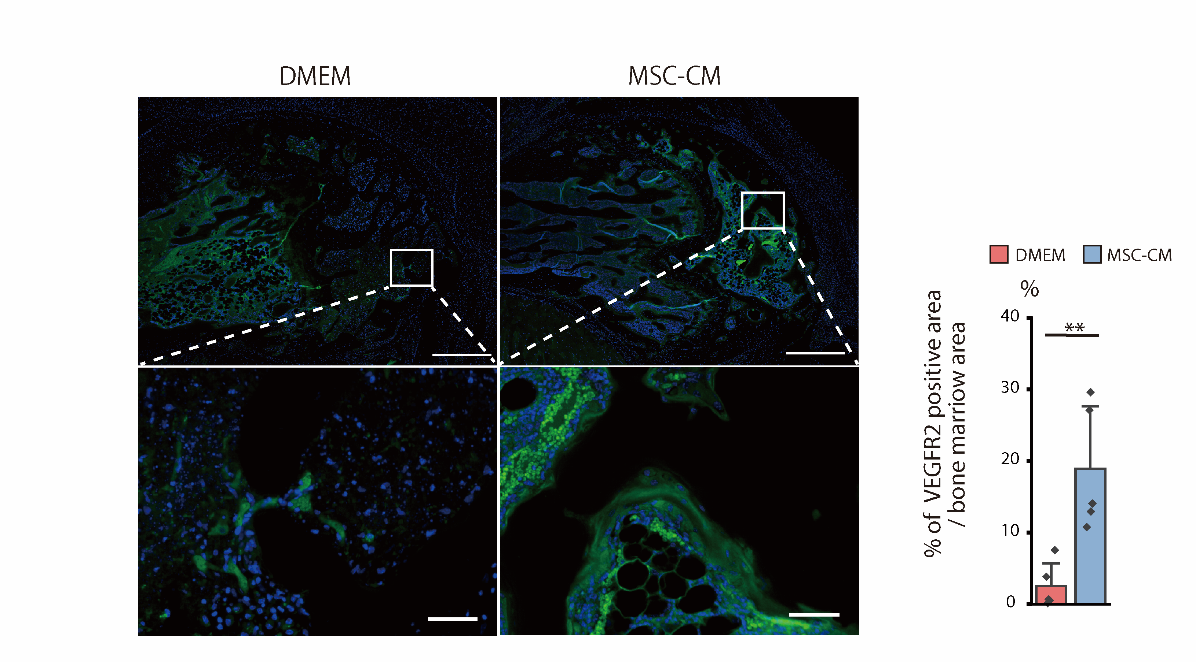


**Supplementary Figure 2. Effects on MSC-CM in promoting bone marrow revascularization 2 wks after ischemic osteonecrosis induction.** Representative images of fluorescent immunostaining for VEGFR2 (ab194806; Abcam) in the distal femur 2 wks post-surgery are shown. Scale bars = 500 μm (top) and 50 μm (bottom). The VEGFR2 signal in the bone marrow at the distal end was quantified. The proportions of VEGFR2-positive areas in the bone marrow area are shown in the graph. The MSC-CM group had significantly more areas of VEGFR2 expression in their epiphyseal bone marrow than did those of the DMEM group (18.9 ± 7.8% vs. 2.5 ± 2.8%; p = 0.004). Data consists of a dot plot and the mean ± standard deviation. **P < 0.01 by unpaired, two-tailed Student’s *t*-test. (n = 5/group).


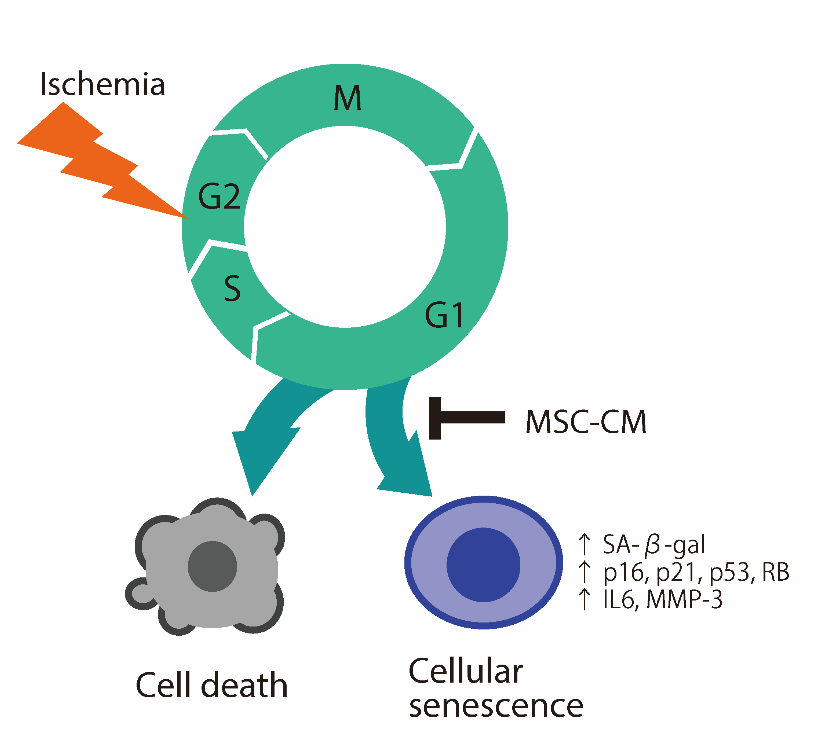


**Supplementary Figure 3. Schematic image showing the suppression of cellular senescence using mesenchymal stem cell-conditioned medium (MSC-CM).** Damage caused by ischemia results in cell death or cellular senescence. Senescent cells express senescence-associated-β-galactosidase and senescence-associated genes and show a senescence-associated secretory phenotype. Administration of MSC-CM prevents cellular senescence, although does not prevent cell death.

**Supplementary Table.S1**

| primer sequences used in qRT-PCR | | |  |
| --- | --- | --- | --- |
| primer |  | sequence forward (5'-3') | sequence reverse (3'-5') |
| human |  |  |  |
|  | p16INK4a | CCCCGATTGAAAGAACCAGAGA | ACGGTAGTGGGGGAAGGCATAT |
|  | p21 | CCGCCCCCTCCTCTAGCTGT | CCCCCATCATATACCCCTAACACA |
|  | p53 | CCGGCGCACAGAGGAAGAGA | TGGGGAGAGGAGCTGGTGTTGT |
|  | RB | TTCCCAGGTTCTGTTTAT | GTTATCAGGACTCCCACT |
|  | RANKL | GCCTTTCAAGGAGCTGTGCAAAA | GAGCAAAAGGCTGAGCTTCAAGC |
|  | IL-6 | GGTACATCCTCGACGGCATCT | GTGCCTCTTTGCTGCTTTCAC |
|  | MMP-3 | GGGCCATCAGAGGAAATGAG | CACGGTTGGAGGGAAACCTA |
|  | GAPDH | CTTTGGTATCGTGGAAGGACTC | GTAGAGGCAGGGGATGATGTTCT |
| mice |  |  |  |
|  | p16INK4a | GAACTCTTTCGGTCGTACCC | CGAATCTGCACCGTAGTTGA |
|  | p19 | TGAGGCTAGAGAGGATCTTGAG | CAGAAGAGCTGCTACGTGAA |
|  | p21 | AGGCAGACCAGCCTGACAGAT | TCCTGACCCACAGCAGAAGAG |
|  | p53 | GTTGCTGCCCCAGGATGTTG | ATGACAGGGGCCATGGAGTG |
|  | RB | TTCCACTCGAACACGAATGC | TCACAACCATGAGCCAGGAG |
|  | IL-6 | GAGGATACCACTCCCAACAGACC | AAGTGCATCATCGTTGTTCATACA |
|  | MMP-3 | TTGACGATGATGAACGATGGA | GAGCAGCAACCAGGAATAGGTT |
|  | BMP2 | GGGACCCGCTGTCTTCTAGT | TCAACTCAAATTCGCTGAGGAC |
|  | RANKL | TCCGAGCTGGTGAAGAAATT | GAGAGGGCTGTGAGTTTCAT |
|  | DKK1 | CAGTGCCACCTTGAACTCAGT | CCGCCCTCATAGAGAACTCC |
|  | sclerostin | AGCCTTCAGGAATGATGCCAC | CTGGAGGGTATTGAAGAGCCG |
|  | GAPDH | ACCCCTTCATTGACCTCAAC | TCCCGTTGATGACAAGCTTC |
